# Synthesis and Characterisation of ETS-10/Acetate-based Ionic Liquid/Chitosan Mixed Matrix Membranes for CO_2_/N_2_ Permeation

**DOI:** 10.3390/membranes4020287

**Published:** 2014-06-19

**Authors:** Clara Casado-Coterillo, María del Mar López-Guerrero, Ángel Irabien

**Affiliations:** 1 Department of Chemical and Biomolecular Engineering, Universidad de Cantabria, Avda. Los Castros s/n, 39005 Santander, Spain; E-Mail: irabienj@unican.es; 2 Department of Analytical Chemistry, Faculty of Sciences, Universidad de Málaga, 29071 Málaga, Spain; E-Mail: mmlopez@uma.es

**Keywords:** mixed matrix membranes, chitosan, microporous titanosilicate ETS-10, 1-ethyl-3-methyl-imidazolium acetate ionic liquid, membrane characterisation

## Abstract

Mixed matrix membranes (MMMs) were prepared by incorporating organic surfactant-free hydrothermally synthesised ETS-10 and 1-ethyl-3-methylimidazolium acetate ionic liquid (IL) to chitosan (CS) polymer matrix. The membrane material characteristics and permselectivity performance of the two-component membranes were compared with the three-component membrane and the pure CS membrane. The addition of IL increased CO_2_ solubility of the polymer, and, thus, the CO_2_ affinity was maintained for the MMMs, which can be correlated with the crystallinity, measured by FT-IR, and void fraction calculations from differences between theoretical and experimental densities. The mechanical resistance was enhanced by the ETS-10 nanoparticles, and flexibility decreased in the two-component ETS-10/CS MMMs, but the flexibility imparted by the IL remained in three-component ETS-10/IL/CS MMMs. The results of this work provide insight into another way of facing the adhesion challenge in MMMs and obtain CO_2_ selective MMMs from renewable or green chemistry materials.

## 1. Introduction

The separation and capture of CO_2_ from flue gas is becoming important for greenhouse emission control and strong demand of more energy-, cost-effective and environmentally friendly technologies are growing. Membrane-based gas separation has been postulated to compete with absorption in terms of energy requirement when CO_2_ content in the feed is larger than 20%, as in cement or steel factories [1], due to its low energy consumption, easy operation, and low maintenance.

The membrane is the key element since the separation phenomenon occurs in it, thus, the material used in the membrane preparation determines the separation performance. Depending on the material, membranes are usually classified as polymeric, inorganic, and, more recently, mixed matrix membranes (MMMs). Transport through a dense-polymeric membrane usually takes place through the solution-diffusion mechanism in three steps: (i) the selective component adsorbs in the membrane; (ii) where diffuses through; and (iii) the component desorbs from the other side, due to the low pressure kept at the permeate side. Commercial polymer membranes are relatively easily processed at low costs, but their limited resistance to high temperature, usual inadequacy to high flow rates, or sensitiveness to clogging by dust, there is an absence of economy of scale and low selectivity to CO_2_/N_2_ separation [2]. Inorganic membranes have good catalytic and separation behaviour and present good chemical and temperature resistance. The transport mechanism depends usually on the pore size distribution of the selective layer and, although there are several inorganic membranes commercially available for pervaporation and vapour permeation and liquid filtration processes, not yet for gas separation [3]. The reproducibility and fabrication cost is still a major challenge [4]. 

**Figure 1:**
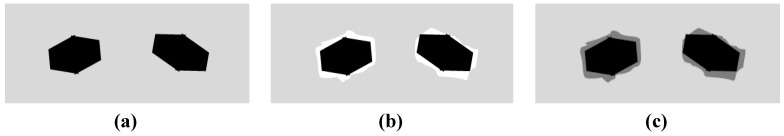
Schemes of the most typical nanoscale morphologies in MMM structures adapted for ETS-10 in the chitosan polymer membrane: ideal morphology (**a**); “sieve-in-a-cage” presence of interfacial voids (**b**); and rigidification by free volume reduction (**c**).

Morphology issues have been addressed in many different membrane materials, from pure zeolite membranes [5], to hollow fibre [6], or composite membranes [7]. MMMs are heterogeneous materials formed by the combination of an organic polymer continuous matrix and inorganic material dispersed phase, with the aim of obtaining a well-dispersed heterogeneous mixture of synergistic properties and overcoming the accepted trade-off between permeability and selectivity for gas separation membranes [8,9]. Permeability is a unit flux defining the gas molecules a membrane lets go through, normalized by operation factors such as pressure and thickness. Thus, permeation takes place because of a chemical potential difference across the membrane, which means that the driving force is usually partial pressure difference. To simplify the understanding of the system, the permeability is calculated as the product of solubility (*S*) and diffusivity (*D*). The solubility coefficient is given by the condensability of the penetrant, *i.e*., CO_2_ in the case of the present work, in membrane material, and the diffusivity coefficient depends on the size and shape of CO_2_, the free volume or porosity and pore size distribution and the chain flexibility of the polymer. Adhesion between dispersed and continuous phases is the main challenge in MMMs, leading to several morphologies as those represented in Figure 1, thus affecting separation performance. Figure 1a is the schematic representation of the ideal morphological contact among inorganic fillers and polymer. Figure 1b refers to the non-ideal detachment between inorganic fillers and polymer matrix, creating voids around the former that alter the gas perm-selectivity performance. Figure 1c corresponds to the non-ideal free volume reduction by polymer chain rigidification and pore blockage. In order to improve adhesion, several strategies have been studied, such as functionalization of the inorganic particles prior to introduction into the polymer matrix, or the incorporation of an ionic liquid in the membrane matrix [10,11]. The novelty of this work consists in attempting a similar approach but using a non-toxic ionic liquid, a biopolymer, and a microporous zeo-type material prepared without costly organic surfactant.

The continuous polymer chosen is chitosan (CS), poly[β(1→4)-2-amino-2-deoxy-d-glucopyranose], a linear polysaccharide obtained by the deacetylation of chitin, an abundant natural polymer, cheap and obtained from renewable sources, *i.e*., the shell of crustaceans. CS is biodegradable, biocompatible, non-toxic, and hydrophilic. The high hydrophilicity of chitosan makes it prone to hydrate and form water-swollen membranes with enhanced CO_2_:N_2_ perm-selectivity because of the high CO_2_ solubility in water [12]. Swollen CS-based membranes have been studied for CO_2_ separation, usually by humidifying the feed gas prior to entering the membrane module [13] for CO_2_:N_2_ 50:50 (vol %) mixtures and recently the influence of free and bound water on separation performance was analysed, for diluted CO_2_:N_2_:H_2_ mixtures simulating flue gas streams [14,15]. Its mechanical stability has, nevertheless, been tried to improve by coating on a porous polysulfone support [16], organic chemical crosslinking [17], and physical mixing with zeolite particles [18]. Facilitated transport in the solid matrix is expected to increase the stability as well, and CS, because of the weak acid-base interactions between CO_2_ and water molecules and the amino groups in the chains, has potential to enhance the electrostatic interactions among permeating molecules and the functional groups in the polymer by introducing appropriate materials.

The structure of the microporous titanosilicate ETS-10 is made of orthogonal TiO_6_ octahedra and SiO_2_ tetrahedra linked by oxygen atoms shared in the corners. Ti atoms in a six-coordinated state have two negative charges balanced by Na^+^ and K^+^ [19]. The high cation exchange capacity is what makes ETS-10 very interesting in adsorption [20], catalysis, and membrane separation processes [21]. ETS-10 can be synthesised in different sizes including nano-scale [19], which may be homogeneously dispersed in a polymer providing this with its intrinsic characteristics.

Ionic liquids (ILs) combining good and tuneable solubility properties with negligible vapour pressure and good thermal stability have recently received much attention as green solvents and CO_2_ absorbents in supported liquid membrane contactors [22]. The CO_2_ solubility is higher when acetate is the anion and the shorter length of the cation, and 1-ethyl-3-methylimidazolium acetate, [emim][Ac], the room temperature ionic liquid (IL) with the highest reported CO_2_ solubility [23], as well as non-reported toxicity [24], was chosen for the proof-of-concept of this work. A good interaction with CS is expected since it has been reported as a good solvent for polysaccharides [25], because of the strong H-bonds forming with the OH groups in the polymer chain. CS and chitin have been reported to enhance the CO_2_ solubility of low absorbing [bmim][Cl] because the ionic liquid is able to alter the H-bonds in the polymer chains, thus freeing amino groups that become available for CO_2_ absorbing sites [26]. 

Table 1 shows the chemical formula and properties of all the components.

**Table 1:** Main properties of the mixed matrix membranes (MMM) components used in this work.

Name	Molecular Formula	Properties
1-Ethyl-3-methylimidazolium acetate, [emim][Ac] (IL)	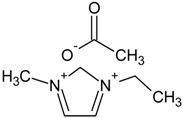	Density = 1.03 g/cm^3^ [23]
Chitosan (CS)	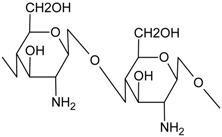	Density = 0.942 g/cm^3^ [27] Δ*H*_m_ (J·g^−1^) = 334.4 [28]
ETS-10	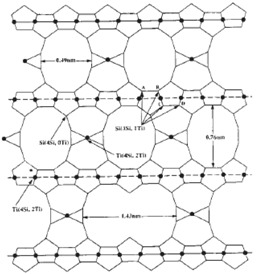	Density = 1.75 g/cm^3^ [29]

In this work, novel MMMs composed of CS, IL/CS, ETS-10/CS and ETS-10/IL/CS, with the small loading of dispersed filler of 5 wt % with respect to the continuous polymer matrix, were prepared and tested for CO_2_ and N_2_ permeation. Factors affecting membrane morphology such as adhesion, interaction among components, and thermal, chemical, and mechanical resistance have been explored and discussed.

## 2. Results and Discussion

### 2.1. Thermal Properties

**Figure 2:**
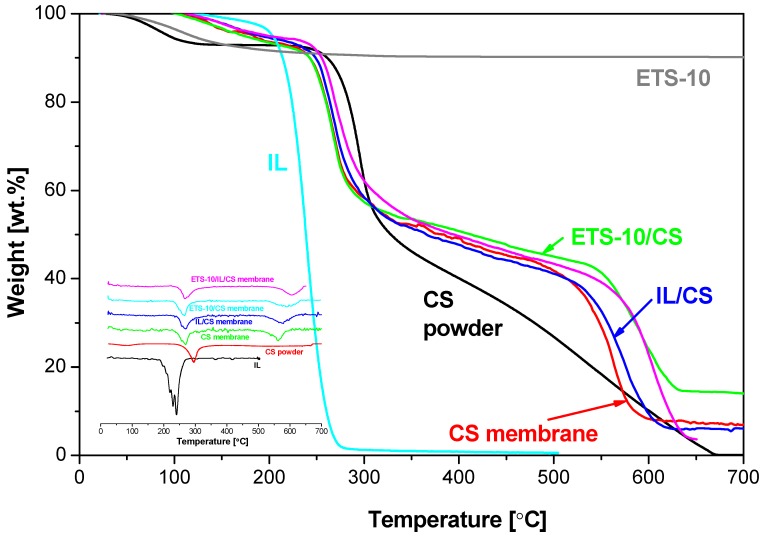
TGA curves of the CS and CS-hybrid membranes. TGA of CS powder, ETS-10 crystalline particles and IL are also included for comparison. DTG curves are presented in the inset.

The thermal properties of the MMMs are shown in the TGA diagrams in Figure 2. The major weight loss for all the materials involved in membrane preparation occurs at a temperature between 250 and 630 °C. This weight loss is due to the decomposition of the matrix. The thermal decomposition of the IL occurs at 200 °C, which agrees with literature [23]. The decomposition of chitosan powder (560 °C) agrees with literature [30], where the degradation of CS is established in two stages and decomposition temperature is considered at the end of the first stage (540 °C). Regarding the CS-based membranes prepared in this work, the pristine CS membrane has almost the same decomposition temperature as the precursor chitosan powder (560 °C), which is increased to more than 620 °C upon introduction of ETS-10 inorganic particles, even at the low loading of 5 wt % used in this work. This agrees with the decomposition peak of the second stage that is observed at 564, 579, 593, and 604 °C for the CS, IL/CS, ETS-10/CS, and ETS-10/IL/CS membranes, respectively, but not observed for the CS powder. 

The shifting of the degradation temperature of the CS membranes upon addition of IL and ETS-10 is an indication of enhanced thermal stability, which may be of the potential of these novel membrane materials for higher temperature CO_2_ separation.

**Figure 3:**
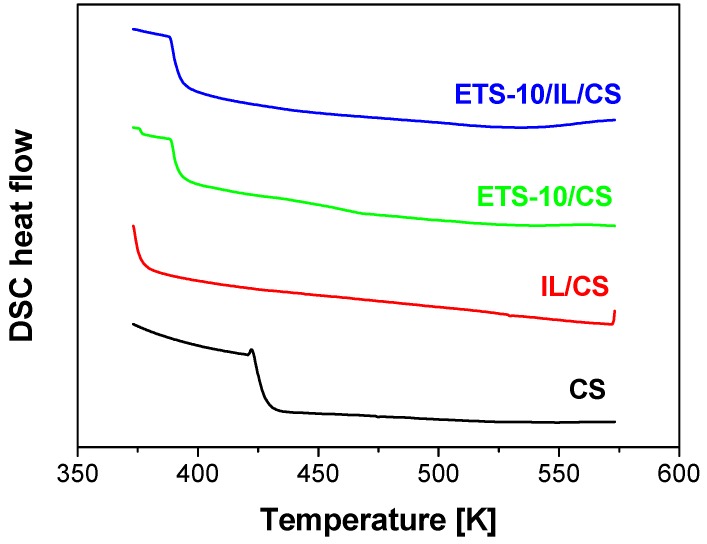
DSC plots of CS-based membranes.

Figure 3 shows the DSC plot of the CS-based membranes prepared in this work, which is the usually reported technique to provide information regarding change from rubbery to glassy state in a polymer material, and this is the method for estimating the crystallinity of semi-crystalline polymers. In Figure 3, only ETS-10/CS MMM allows discerning a glass transition temperature around 196 °C, which agrees with previous observations [18], because the glass transition of CS is generally reported difficult to discern by DSC because of the huge hydrophilicity [17].

### 2.2. Mechanical Properties

Table 2 collects the mechanical properties of the CS and MMMs studied in this work. The tensile strength of pure CS membranes agrees with values reported in literature [31], despite differences on the precursor CS powder employed and membrane preparation protocols, which account from the natural origin of this material. 

**Table 2:** Conditions of the tensile tests (General conditions: sample width 5 mm, grip to grip distance 40.20 ± 8.76 mm).

Membrane Material Composition	Thickness (μm)	Filler Content (wt %)	Tensile Strength (MPa)	Elongation at Break (%)
CS	121.9 ± 3.96	0	31.63 ± 7.41	18.52 ± 8.23
IL/CS	128.0 ± 3.57	5	16.09 ± 11.04	40.44 ± 12.45
ETS-10/CS	130.0 ± 4.50	5	24.30 ± 4.88	14.40 ± 9.38
ETS-10/IL/CS	168.0 ± 5.0	5 (each)	19.93 ± 5.01	36.15 ± 3.03

The tensile strength diminishes upon addition of IL and ETS-10 particles. This was attributed to plasticisation of the polymer matrix reflected by the large increase on the value of the elongation at break, for the IL/CS with respect to CS membranes. The introduction of [bmim][CF_3_SO_3_] IL in semi crystalline Pebax polymers [32] caused a decrease in elongation at break that has not been observed here. On the other hand, the elongation at break of the CS based membranes decreases upon addition of ETS-10 particles. This is due to the rigidification of the organic polymer [33] by the addition of inorganic fillers. 

### 2.3. Structural and Morphological Properties

**Figure 4:**
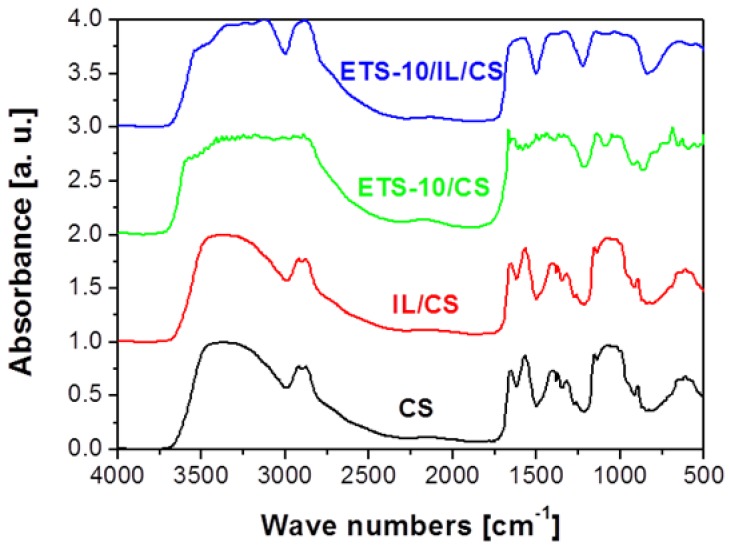
FT-IR measurements of CS-based membranes.

The increased flexibility imparted to the MMMs by the introduction of the IL was attributed to the singular interaction between CS and IL, and that was the reason to measure FT-IR spectra of the membranes (Figure 4). The CS and 3-component ETS-10/IL/CS membranes demonstrated a broad band in the range 3600–2700 cm^−1^, attributed to NH and OH vibrations, whereas the broad band at around 2940 cm^−1^ in CS is shifted to lower wave numbers due to film formation. In the second region, bands attributed to amide I (1633 cm^−1^), amide II (1537 cm^−1^) and amide III (1315 cm^−1^) in CS [34], decreased in intensity, and are mixed up with the microporous titanosilicate ETS-10 and 1-ethyl-3-methyl-imdazolium acetate IL own bands upon hybridisation. This confirms the good interaction existing among the components involved on MMM preparation, and that may account for the higher flexibility of the hybrid membrane materials imparted to both CS and ETS-10/CS MMMs, as explained above regarding mechanical properties of the membranes in Table 2.

The crystallinity, χ, of the CS-based membrane samples was calculated from the FTIR spectra, using the ratio of the absorbance at 1423 and 890 cm^−1^, respectively. The values are collected in the last column of Table 3, together with other morphological properties of the membranes. The crystallinity increased monotonously upon addition of IL, ETS-10 and both to the CS polymer matrix, as reported for other MMMs prepared from semi crystalline polymers [35], which attribute this phenomenon to the role of inorganic fillers as nucleating agents. The crystallinity is important in membrane analysis since the amorphous part of the polymer is the main contributor to the gas transport.

**Table 3:** Morphological properties of CS-based membranes: Theoretical density (ρ_add_), measured density (ρ_m_), void volume fraction (ϕ_v_) and true dispersed filler volume fraction.

Membrane materials	ρ_add_ (g/cm^3^)	ρ_m _ (g/cm^3^)	ϕ_v_ (vol/vol)	ϕ_d_ (vol/vol)	χ (-)
CS	0.942	0.727 ± 0.26	0.228	0.520	0.14
IL/CS	0.941	1.108 ± 0.48	−0.165	0.032	0.18
ETS-10/CS	1.014	0.637 ± 0.14	0.372	0.028	0.28
ETS-10/IL/CS	1.442	0.714 ± 0.14	0.505	0.304	0.34

The theoretical density (ρ_add_) of the membrane was calculated using the additive model, as follows,

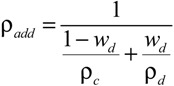
(1)
where *w*_d_ is the weight fraction of the dispersed phase (IL or ETS-10) in the continuous matrix, ρ_d_ and ρ_c_ are the densities of the dispersed and the polymer phase in the continuous matrix in Table 1. 

The nominal volume fraction of the filler, 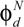, is described as:

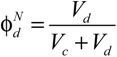
(2)
where *V*_d_ and *V*_c_ are the ideal contributions of the dispersed and continuous phases, the inorganic filler and organic continuous matrix, respectively, to the total volume, defined as the mass of the organic and inorganic components added to the composite, divided by the pure polymer or filler density. In this work, the CS polymer or IL/CS hybrid are considered as continuous matrix for the 2- and 3-component MMMs, respectively.

The measured density values are always smaller than the theoretical values. This accounts for the void fraction created in the MMM upon addition of the porous ETS-10 nanoparticles, which resulted in lower density. This is the reason why the ideal MMM morphology, where a perfect adhesion between the organic and the inorganic phase, as depicted in Figure 1a, is seldom present in reality and the non-ideal free-volume in Figure 1b, attributed to poor interfacial contact between dispersed and continuous phases (Figure 1c), must be taken into account. The true filler volume fraction, ϕ_d_, was calculated by comparing the experimental and the theoretical densities, when the void volume fraction can be calculated as [35]:

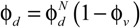
(3)

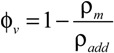
(4)

The void fraction increased significantly for the two-component ETS-10/CS and the three-component ETS-10/IL/CS MMMs above that of CS membranes. This increase can be attributed to several factors: polymer crystallinity, interphase voids between the particles and the porosity of the crystals themselves [35,36]. The highest value of the density for the IL/CS membrane and the negative value for the void volume fraction may be attributed to the intimate contact between IL and CS. 

Table 4 summarises the solubility values calculated from thermo gravimetrical sorption experiments. It is worth noticing that the solubility values of the single components do not agree with the additive rule, which correlates with the observation of non-idealities and void fraction calculations in Table 3, in agreement with the MMM morphologies in Figure 1. The CO_2_ solubility increases upon addition of ETS-10 particles, which agrees with our assumption that the incorporation of this alkaline titanosilicate favours CO_2_ permeation due to its adsorption affinity toward CO_2_. The N_2_ solubility decreased about 42% in ETS-10/CS MMMs, which was attributed to the molecular sieve effect of the nanoporous titanosilicate. The average pore opening of ETS-10 is 0.8 nm [37]. The N_2_ solubility in the three-component ETS-10/IL/CS MMMs was further reduced, due to the incorporation of the highly CO_2_-soluble IL, while CO_2_ solubility remained constant, compared with the two-component ETS-10/CS MMM. This accounts for the CO_2_ affinity of the novel membrane materials synthesized in this work.

**Figure 5:**
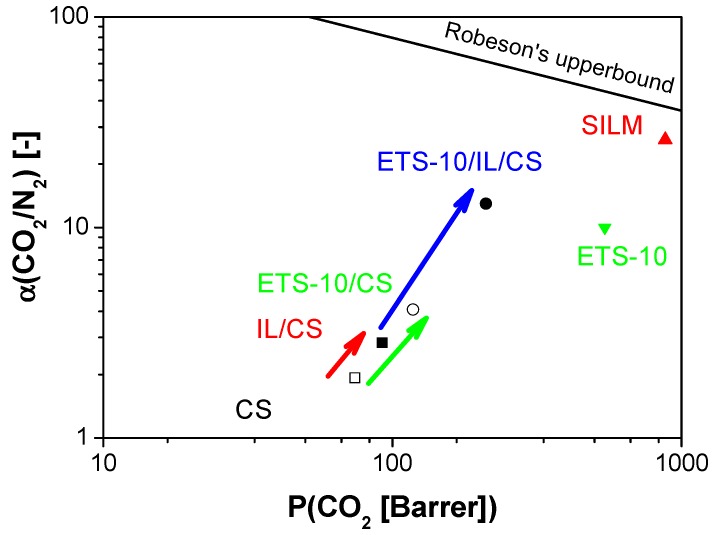
Comparison of CO_2_ permeability and CO_2_/N_2_ selectivity for the CS-based MMMs with Robeson’s upper bound [8A]. Void symbols represent the membranes where CS is the continuous phase, filled symbols those where the continuous phase is constituted by IL/CS. Dense CS, IL, and ETS-10 are literature pair values for dense CS membranes [38], supported [emim][Ac] membranes on a porous PVDF disk [22] and pure ETS-10 inorganic membranes on porous alumina supports [21], respectively.

The permeation data obtained experimentally in this work are plotted against the Robeson’s upper bound in Figure 5. There is a monotonous increase in permeability and selectivity, as the pure CS matrix is hybridized by the introduction of the small amounts of IL, ETS-10, and both. The highest permeability of the ETS-10/IL/CS MMMs is due to the IL/CS continuous phase and interaction of the IL both with particles and polymer [11]. The loading amount is kept constant as low as 5 wt % with respect to CS concentration, and there is no decrease in selectivity observed, the MMM performance almost reaching that of other membrane morphologies, such as IL supported membranes (SILM) and purely inorganic ETS-10 tubular membranes.

**Table 4:** Solubility (*S*) of measured gases in MMMs.

Membrane material	*S*(CO_2_), cm^3^ (STP)/cm^3^ cmHg	*S*(N_2_), cm^3^ (STP)/cm^3^ cmHg	Solubility Selectivity (CO_2_/N_2_)
IL	0.0114	- ^a^	- ^a^
ETS-10	0.1019	- ^a^	- ^a^
CS	0.0767	0.0050	15.34
IL/CS	0.5065	0.0255	19.84
ETS-10/CS	0.0798	0.0029	27.58
ETS-10/IL/CS	0.0724	0.0019	38.48

^a^ Lower than detection limits.

Hence, the addition of IL in just a small amount in the membrane not only increased permeability, but also seemed to act as intermediate medium imparting improved adhesion between the CS continuous polymer and the ETS-10 dispersed nanoparticles as well as enhanced CO_2_ affinity.

## 3. Experimental Section

CS (coarse ground flakes and powder, Sigma-Aldrich) with a deacetylation degree higher than 75 wt % and high viscosity in 1 wt % acetic acid/water was used as purchased. This CS product provides high density of amino groups for CO_2_ separation [38]. 

The ETS-10 nano-crystals were prepared according to a hydrothermal synthesis method previously reported [16,39] using TiO_2_-anatase (powder, 99.8 wt %, Aldrich, Madrid, Spain) and sodium silicate solution (27 wt % SiO_2_, 8 wt % Na_2_O, Merck, Barcelona, Spain) as Ti and Si source, respectively. In a typical synthesis, 35.06 g of parent gel with molar composition 5.6 SiO_2_:1 TiO_2_:4.6 Na_2_O:1.9 K_2_O:137 H_2_O were poured into a Teflon-lined autoclave and submitted to hydrothermal synthesis at 230 °C for 24 h. The autoclave was then removed from the oven and quenched under cold tap water to room temperature. The solid was washed and centrifuged at least 3 times, and dried at 100 °C overnight to recover about 2.8 g of final product. This product has a particle size of *a* = *b* = 0.32 ± 0.06 µm and *c* = 0.41 ± 0.22 µm and a BET surface area of 253 ± 7 m^2^/g [19].

### 3.1. Membrane Preparation

The procedure to prepare each type of CS MMM is as follows. First, CS 2 wt % solutions were first dissolved in 2 wt % acetic acid (glacial, Panreac) aqueous solutions under stirring at 80 °C for 24 h at reflux conditions. The CS solution obtained was filtered to remove insoluble impurities and degassed in an ultrasonic bath before 10 mL on a polystyrene Petri dish and evaporating at room temperature for 2–3 days. CS membranes were then removed from the Petri dish. A 15.55 cm^2^ membrane was cut from the film for gas permeation and neutralized in 1 M NaOH and rinsed with abundant distilled water and dried at 4 °C before CO_2_ and N_2_ permeation experiments in order to ion-exchange the NH_3_^+^ functional groups of the polymer matrix. Excess water was carefully removed by blotting the membrane, for diluted CO_2_:N_2_:H_2_ mixtures simulating flue gas streams [14,15]. Its mechanical stability has nevertheless been tried to improve by coating on a porous polysulfone support [16], organic chemical crosslinking [17], and physical mixing with zeolite particles [18]. Facilitated transport in the solid matrix is expected to increase the stability as well, and MMM with 5 wt % ETS-10 loading were prepared as reported elsewhere [18]. ETS-10 particles were first dispersed in distilled water (proportion 1:100 wt/wt) in an ultrasound bath for 10 min at room temperature. Then, CS solution (10 mL) was added and treated in ultrasound bath for 15 min until a homogenous white dispersion was obtained and cast as described above.

IL/CS membranes were prepared with a nominal 5 wt % IL loading with respect to CS. In a typical synthesis: 0.042 g of IL (97 wt %, Sigma-Aldrich, Madrid, Spain) were added to the 10 mL CS solution and stirred overnight before casting in a similar manner as the pure CS membranes.

For the three-component ETS-10/IL/CS MMMs, the preparation method was similar to that employed for ETS-10/CS MMMs, using the IL/CS mixture as continuous phase. 

### 3.2. Characterization Methods

Thicknesses were measured using a Mitutoyo Digital Micrometer with an accuracy of 0.001 mm after removal from the Petri dishes, measured in five points covering the whole membrane surface, before NaOH neutralisation treatment. The thicknesses of selected membranes were measured before and after the permeation measurement in order to check the validity of these values and the structural stability of the membranes upon permeation.

The experimental density values of the membranes (ρ_m_) were measured gravimetrically from the electronically measured weight of the circular film and the calculated volume. 

Thermo gravimetric analyses (TGA) were performed in a DTG 60H Shimadzu instrument (Japan) in air from 25 to 700 °C at a heating rate of 10 °C/min, in order to study the thermal stability of the resulting membranes. The decomposition temperature was calculated as the temperature at which 5% weight loss occurs. The gas solubility of the membranes was evaluated by CO_2_ and N_2_ adsorption measured gravimetrically at the same thermo balance mentioned above, which is equipped with a FC60A flowmeter (Shimadzu, Japan). The sorption experiments were conducted isothermally at 25 °C for 3 and 4 h, under CO_2_ and N_2_ flow, respectively and a gas pressure of 5 bar. DSC analyses were carried out in a DSC 822 apparatus from Mettler Toledo, belonging to the Universidad de Zaragoza (Zaragoza, Spain). The samples were heated at 10 °C/min from 100 to 300 °C, after 1 min at 100 °C, twice, to elucidate the glass transition in the second run.

The mechanical resistance of the membranes was measured by the tensile strength and the elongation at break of 5–10 of 5 mm wide samples of the membrane materials in a Universal Testing Machine (Zwick/Roell, Ulm, Germany) with a head load up to 2.5 kN and 5 mm/min. 

IR spectra were recorded on a Perkin Elmer Spectrum 100 FTIR spectrometer (Concord, Canada) with a resolution of 4 cm^−1^ and 32 scans, at the Universidad de Málaga (Málaga, Spain). The solid samples were measured after dilution in KBr pellets. For the membrane samples, these were dried at 100 °C for at least 2 h and grinded for 5 min prior to pellet preparation. The IL sample was sandwiched drop wise into two thin pieces of glass and measured using an optical transmission cell. All windows used were planar and compatible with infrared wavelengths. 

Gas permeation was carried out with pure N_2_ and CO_2_ at room temperature in a constant volume system. Membranes were placed in the permeation cell, and tested for N_2_ first and then CO_2_. An average of 10 experimental runs were performed for each membrane composition. The experimental runs were left no more than 3 h in order to keep the membrane at constant operation conditions (relative humidity, pressure and driving force), and only the results obtained for the membranes that were still flexible when removed from the module were taken into account. In a typical run, gas was fed to permeate and feed compartments, which took a few minutes. Then, gas feed was closed and the vent valve was opened to empty the permeate compartment and generate the driving force across the membrane. Initial pressure was measured by a pressure transducer (Omega, Manchester, UK), and the pressure difference was monitored along the experimental run by a differential pressure transducer (Omega, Manchester, UK). The steady-state permeability (1 Barrer = 10^−10^ cm^3^ (STP) cm cm^−2^ s^−1^ cmHg^−1^) was calculated from the steady-state flux. Ideal selectivity α(CO_2_/N_2_) is defined as the ratio of the permeability values of the two gases, the faster gas permeability (CO_2_) divided by the slower gas permeability (N_2_), thus constituting an intrinsic property to compare different membrane materials.

## 4. Conclusions

Mixed matrix membranes (MMMs) were prepared by adding a highly absorbing non-toxic ionic liquid, 1-ethyl-3-methylimidazolium acetate ([emim][Ac], IL), and a microporous titanosilicate ETS-10 nanoparticles to the continuous matrix of biopolymer chitosan (CS), and neutralising in NaOH solution to prepare the materials for CO_2_ affinity. The membrane material characteristics and permselectivity performance of the two-component membranes was compared with the three-component membrane and the pure CS membrane. The CO_2_ solubility increased upon addition of the IL and ETS-10 adsorbent. The mechanical strength was enhanced by the addition of ETS-10 particles, while the IL imparted flexibility to the membrane. The porosity and free volume were estimated from measured and theoretical densities. The void fraction of the ETS-10/IL/CS MMM was the largest obtained in this work, attributed to the interaction between IL, the polymer, and the porous ETS-10 particles. FT-IR spectra revealed a good interaction between the components in the MMMs, and a means of measuring the crystallinity to account for the real permeation area of the membrane, which is usually the amorphous part. These parameters (pore size, particle size and shape, interfacial adhesion, free volume, crystallinity) were correlated with the gas transport performance. This was evaluated by CO_2_ and N_2_ single gas permeation experiments. The selectivity of the three-component ETS-10/IL/CS MMMs was six times higher than pure CS membrane, showing in general a positive trend towards the attractive region of Robeson’s upper bound.
